# 

**Published:** 1862-01

**Authors:** 


### HALL'S JOURNAL OF HEALTH.

Vol. 9. JANUARY, 1862. No. 1.

### HEALTH TRACTS.

The January and February numbers contain sixty-two one-page Health Tracts. The number of the tract answers to the number of the page. January contains from No. 1 to 31, both included; February has No. 32 to No. 62, both included.

| | Tract |
|---|---|
| Air and Sunshine, . . . . | 51 |
| Apples, . . . . . . . . | 59 |
| Burns and Bites, . . | 27, 23 |
| Baths and Bathing, . . . | 30 |
| Colds Cured, . . . . | 27, 3 |
| Colds Neglected, . . . | 27, 29 |
| Colds Prevented, . . . . | 31 |
| Corns and Shoes, . . . . | 14 |
| Consumption and Measles, | 11, 16 |
| Coffee Drinking, . . . . . | 46 |
| Catarrh, . . . . . . . . | 52 |
| Checking Perspiration, . . | 58 |
| Costiveness, . . . . . . . | 22 |
| Drunkenness and Dyspepsia, | 8 |
| Dyspepsia, . . . . . . . | 33 |
| Disease, Causes of, . . . . | 4 |
| Disease Avoided, . . . . | 28 |
| Drinking, . . . . . . . . | 35 |
| Diet for Invalids, . . . . | 54 |
| Erect Position, . . . . . | 11 |
| Eating, . . . . . . . . | 26 |
| Eating Wisely, . . . . . | 32 |
| Eat, How to, . . . . . . | 34 |
| Eating, When and What, . | 40 |
| Eyes, Care of, . . . . | 5, 15 |
| Eyesight Failing, . . . . | 61 |
| Erysipelas, . . . . . . . | 56 |
| Fruits in Summer, . . . . | 2 |
| Flannel, Wearing, . . . | 19 |
| Feet, Care of, . . . | 14, 21 |
| Fifteen Follies, . . . . . | 53 |

| | Tract |
|---|---|
| Growing Beautiful, . . . | 15 |
| Hair, the Care of, . . . | 18 |
| Headache, . . . . . . | 41, 62 |
| Health without Medicine, . | 20 |
| Healthful Observances, . . | 38 |
| Health's Three Essentials, | 19, 42 |
| Hydrophobia, . . . . . . | 49 |
| Inconsiderations, . . . . . | 1 |
| Ice, its Uses, . . . . . . . | 9 |
| Music Healthful, . . . . | 7 |
| Medicine, Taking, . . . . | 60 |
| Nothing but a Cold, . . . | 36 |
| Neuralgia, . . . . . . . . | 44 |
| Nursing Hints, . . . . | 57 |
| Precautions, . . . . . . . | 37 |
| Presence of Mind, . . . | 39 |
| Premonitions, . . . . . . | 43 |
| Private Things, . . . . | 45 |
| Poisons, . . . . | 23, 27, 55 |
| Rheumatism, . . . . . . | 50 |
| Sitting, . . . . . . . . . | 13 |
| Sabbath Physiology, . . . | 17 |
| Sour Stomach, . . . . . . | 24 |
| Sleeping, . . . . . . . . | 25 |
| Sick-Headache, . . . | 62, 41 |
| Traveling Hints, . . . . | 6 |
| The Three Ps, . . . . . . | 47 |
| Winter Rules, . . . . . . | 10 |
| Walking, . . . . . . . . | 12 |
| Warnings, . . . . . . . | 48 |
| Valuable Knowledge, . . . | 27 |

